# Lessons learned from co‐designing a high school beach safety education program with lifeguards and students

**DOI:** 10.1002/hpja.664

**Published:** 2022-09-25

**Authors:** William Koon, Robert W. Brander, Dennis Alonzo, Amy E. Peden

**Affiliations:** ^1^ UNSW Beach Safety Research Group UNSW Sydney Sydney NSW Australia; ^2^ School of Biological Earth and Environmental Sciences UNSW Sydney Sydney NSW Australia; ^3^ School of Education UNSW Sydney Sydney NSW Australia; ^4^ School of Population Health UNSW Sydney Sydney NSW Australia

**Keywords:** adolescence, beach safety, coastal, co‐design, drowning, risk, water safety

## Abstract

**Issue Addressed:**

School‐based beach safety education programs are common, but best practice guidance and information on their design and development is limited.

**Methods:**

Researchers, professional ocean lifeguards and students participated in a co‐design process to inform a lifeguard‐delivered, school‐based beach safety education program for a coastal community in New South Wales, Australia. Lifeguards and researchers (experts) provided structured feedback about the program in a survey and facilitated workshop; students (n = 26) aged 11 to 13 years participated in focus group sessions intended to garner in‐depth understanding of their experiences at the beach and knowledge of, and attitudes towards, beach safety.

**Results and Discussion:**

The co‐design process provided a novel and inclusive model for beach safety education program development, and valuable lessons for future efforts. Specifically, student focus groups identified several facets of pre‐teens and younger teenagers' beach experience that prompted revision of the education program, primarily framing of program content and safety messages. Peers are a primary motivator for this age group and students' burgeoning independence emerged as an important theme. While knowledgeable about beach hazards and risks, students conveyed mixed safety attitudes and self‐reported safety practices, highlighting the importance of designing programs to motivate behaviour and promote safe decision‐making vs raising awareness alone. Findings illustrate the value of adopting co‐design processes for all beach safety programs, school or otherwise.

**So What?:**

Beach safety programs may not be delivering information that is needed, wanted or useful. Structured consultation with the priority population must become standard practice in beach safety and drowning prevention education program development.

## 1 INTRODUCTION

Drowning and other types of injuries are a major problem at beaches globally and in Australia.^1^, ^2^ In many locations, governments and other organisations are charged with preventing incidents and ensuring the safety of beach visitors, which includes promoting safe behaviour via educational efforts. However, the beach is a complex environment for safety promotion: dynamic hazards interact with social factors to create risk that varies by person, place and time.^3^

Education is the most frequently recommended coastal safety prevention strategy in the peer‐reviewed literature,^4^ and is likely the most common beach safety promotion activity worldwide.^5^ Specifically, beach safety education programs in schools are recommended in the Australian and global beach safety literature,^6^, ^7^, ^8^ and swimming and lifesaving education in schools is considered a major policy priority for the water safety sector in Australia.^9^ However, little is known about the design, content and effectiveness of these efforts.

Schools present an opportune environment for health‐related education, including injury prevention.^10^, ^11^ Although fatal coastal drowning rates of children and adolescents are low compared to other age groups,^2^ beach safety and drowning prevention programs are common in Australian schools. Most include content on coastal hazards such as rip currents and promote “swim between the flags,” which is the primary beach safety message in Australia. Beach hazard knowledge and survival skills developed at younger ages, frequently in school programs, are foundations for risk assessment and decision‐making as children move through adolescence and into young adulthood—life stages characterised by decreasing parental/adult supervision and increasing risk‐taking behaviour.^12^

There is some evidence that school‐based beach safety education programs increase knowledge for younger children^13^, ^14^ and those in early adolescence.^15^ Tipton et al identified that pre‐teens and younger teenagers are old enough to appreciate water safety messaging^15^ and are the age group before coastal drowning rates begin to increase.^2^ This age group is complex, marked by biological, cognitive and social transitions^16^ that underscore the need for thoughtfully designed education programs that account for and address these life changes. In addition to age, social markers including cultural factors, disability and gender influence the way students experience the beach; intersectional identities affect how one thinks about safety and respond to beach safety promotion efforts.^17^, ^18^ The development of beach safety promotion programs therefore requires a thorough understanding of beach related behaviour and experiences of the population and their attitudes and beliefs towards beach safety.^19^

Research from New Zealand has improved understanding of self‐perceived beach related risk among older teenagers (ages 15‐18),^20^, ^21^ but there is little understanding of how pre‐teens and younger teenagers experience the beach, think about risk and make safety‐related decisions. Further, no published work has included the perspectives and preferences of students in the design of beach safety education programs for this, or any age group.

To address this gap, we engaged in a co‐design process to develop a school‐based beach safety education program for youth aged 11 to 13 years. Our aim was 2‐fold. First, to explore and describe methodological aspects of the co‐design process for the design and delivery of a beach safety education program. This involved the professional lifeguards who deliver the program, beach safety researchers and the students themselves. Second, prompted by the lack of information specific to this age group, we aimed to improve understanding of how students aged 11 to 13 years from an Australian coastal community experience the beach, perceive coastal risks and hazards, and engage with school‐based beach safety promotion programs. We hope this study and the lessons learned in this process will guide future beach safety education development and delivery in schools, ultimately better preparing young people to make safer decisions at the beach, reducing the risk of injury or death for themselves and others.

## 2 METHODS

### 2.1 Study setting, theory and design

This multi‐component mixed‐methods study involved triangulating feedback and recommendations from both beach safety education experts and students to inform a lifeguard‐delivered, school‐based beach safety education program. The program was designed as part of a pilot beach safety education initiative for Year 7 students (ages 11‐13) in the Lake Macquarie City Council (LMCC) region, a coastal community approximately 130 kilometres north of Sydney in New South Wales, Australia. The LMCC lifeguards, in addition to their water rescue and emergency response duties, are experienced beach safety educators who deliver approximately 70 beach safety presentations each year to primary school children (ages 5‐12). This project represents an expansion of LMCC's safety education efforts to high school‐aged adolescents.

The LMCC beach safety program consists of a 45‐minute presentation by two or three professional lifeguards, who co‐teach using a slide deck and demonstration where applicable. The program generally occurs in‐person on school grounds, usually in a school hall or auditorium setting, with 75 to 200 student participants per presentation depending on the school.

The LMCC lifeguards engaged with researchers from the UNSW Beach Safety Research Group at UNSW Sydney in a co‐design process to optimise the new high school education program for Year 7 students aged 11 to 13 years. As a collaborative initiative between academics and stakeholders with diverse knowledge, skills and experience,^22^ this process featured overarching principles of co‐design and action research methodologies common in both public health^23^, ^24^, ^25^ and education.^26^, ^27^, ^28^ Our fundamental aim was to improve beach safety education practice,^26^ and as such, we partnered to structure an inclusive research process that sought input from experts (researchers), practitioners (the lifeguards) and end users (students).^23^

**FIGURE 1:**
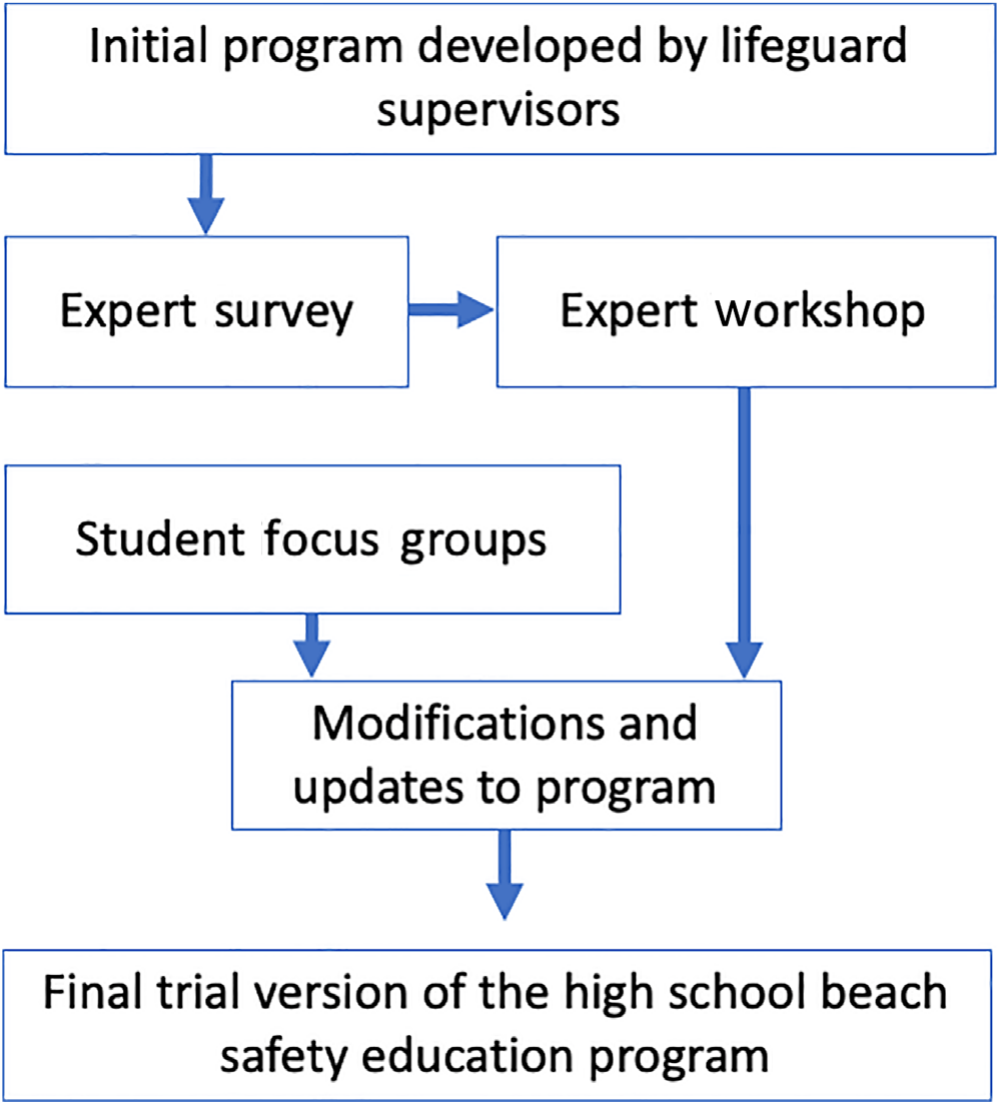
Co‐development process of a school beach safety education program

This process involved three main components, the expert survey, the expert workshop and the student focus groups, which are overviewed here and described in detail in forthcoming subsections (Figure 1). The first version of the program was developed by the LMCC lifeguard supervisor, a veteran lifeguard and experienced safety educator, and was largely based on the existing LMCC primary school beach safety program, adapted for an older adolescent audience. Systematic input on the program's content and design was gathered in an expert survey of beach safety researchers and LMCC professional lifeguards. Results from the expert survey were used to guide a facilitated expert workshop with the lifeguard educators responsible for delivering the presentation in high schools. The pre‐defined purpose of the expert workshop was to allow for further structured input and collective contribution to improving the program. Workshops as a research methodology are suited for this purpose: to produce data about a forward oriented‐process or domain including organisational change and program improvement.^29^

Finally, separate student focus groups with Year 7 students were conducted to garner insight into how this age group experiences the beach and to seek specific input on lifeguard‐delivered presentations in their school. A deeper understanding of experience is important for improving the quality of education programs and services,^24^ and knowledge on adolescent beach experiences including safety perceptions and decision motivators, is limited. Focus groups are useful to this end: they prompt discussion resulting in rich data on feelings, motivations and perceptions^30^ and are helpful for developing and improving health and education programs.^31^

### 2.2 Participants and recruitment

Respondents in the expert survey were researchers from the UNSW Beach Safety Research Group and professional lifeguards from LMCC, recruited via direct email sent from the study team. Participants in the expert workshop were LMCC lifeguards and professional staff involved in coordinating logistics for the program. For both the expert survey and Workshop invitations, potential participants were provided with a description and purpose of the activity and offered alternative formats to provide input to the school program if they wished not to participate.

Participants in the student focus groups were Year 7 high school students who attended a local high school in the LMCC area. Focus groups were organised in partnership with school administrators and took place on campus during a physical education class period. As such, focus group participants were purposefully selected considering the age group and geographic location of interest, accessibility and logistical concerns, and ethical obligations involving student and parent consent.^30^

### 2.3 Data collection and instruments

The expert survey collected responses between 26 April 2021 and 17 May 2021 and consisted of 144 questions. The survey was developed in consultation with the LMCC lifeguard supervisor and designed with a focus on two main domains: program content and slide design. In the survey, respondents were shown images, or videos where applicable, of each slide of the first version of the presentation and asked to provide: (i) a Likert scale rating for content; (ii) a Likert scale rating for design; and (iii) any additional suggestions or comments in an open text box. The survey also included several questions on respondent's general impressions and included space for suggested improvements. Participants used Qualtrics XM software (Version May 2021) to complete the survey during work hours.

The structured expert workshop session took place on 19 May 2021 at a lifeguard office in LMCC, was facilitated by author WK, and lasted approximately 5 hours with breaks. The session was formatted for the group to systematically work through results from the expert feedback survey, considering the data and building consensus for changes to the program. In this sense, the lifeguards as practitioners helped researchers make sense of the survey data and helped arrive at conclusions on what parts of the program to change and how.

Two male (facilitated by WK [male]) and two female (facilitated by AP [female]) student focus groups were held at a high school campus on 9 June 2021 during the students' regularly scheduled 50‐minute physical education class. Discussions followed a semi‐structured interview schedule with key questions relevant to two main domains: student experiences at the beach and their recommendations for beach safety education programs in schools (Supplementary File 1). Age and education may have created a power imbalance where students wanted to provide the “correct” answers; moderators ensured the students at multiple time points in the focus group that there were no right or wrong answers and that students should feel free to express their honest perspectives, experiences and opinions.

Focus group discussions were audio‐recorded with written permission from the students and their parents/guardians; students did not receive incentives for participation and were offered an alternative activity if they did not have their permission slip or chose not to participate. Facilitators (WK and AP) took field notes during and after each focus group discussion for triangulation purposes.

### 2.4 Analysis

From expert survey responses, means and standard deviations were calculated for the Likert questions related to slide content, slide design and overall presentation measures. These were presented alongside qualitative comments and suggestions and served as the basis of the expert workshop. Methods to produce and analyse data from workshops are not well defined^29^; this study adopted a semi‐autoethnography approach that primarily relied on recorded notes from the researcher‐facilitator (WK). This retrospective reconstruction based on secondary data (field notes) provided an account of what happened in the workshop session including: (i) identification of program components that required further development; and (ii) how those components were modified with collective input from the focus group.

For student focus group data, we followed Braun and Clark's six steps for thematic analysis^32^ using guidance from Roberts^33^ and Fereday and Muir‐Charane^34^ for combined deductive/inductive code development; and recommendations from Nowell^35^ to enhance rigour and trustworthiness. Our analysis primarily followed a deductive approach driven by our analytical interests and aims to improve the education program,^32^ but the iterative code development process also included a purposeful inductive component to allow for the development of unexpected themes from the data.^33^ Analyst driven approaches are a feature of action research with a specific purpose, such as improving a program, and have been used in other water safety investigations.^36^

Both WK and AP familiarised themselves with the data via their roles in collection, transcription (WK), writing and reviewing field notes, and from initial transcript readings.^32^ After familiarisation, they pre‐defined an initial set of high‐level codes before commencing the coding process^34^ consistent with recommendations for deductive thematic analysis.^35^ Initial codes were developed using research aims and the Theory of Planned Behaviour, a health promotion framework useful for understanding drivers of human social behaviour and the design of health programs.^37^ Using NVivo (Computer Software), WK and AP independently coded the data to enhance credibility,^35^, ^38^ giving full and equal attention to each data item with specific consideration for nondominant narratives.^32^ The preliminary pre‐defined codes were used as a guide, but the process allowed for development of additional inductive codes to be assigned to segments of data describing a new theme or expanding a pre‐defined code.^34^ During the coding process, WK and AP maintained reflexive journals to track emerging impressions and the development of inductive codes, and met regularly for peer debriefing to discuss the coding process and how thoughts and ideas evolved with further engagement with the data.^35^, ^39^ The process of searching for, reviewing, and defining and naming themes was a reflexive and collaborative processes^32^; both WK and AP iteratively reviewed coded data and discussed discrepancies to evolve thinking and ideas before arriving at a consensus.

### 2.5 Rigour and reflexivity

Researcher positioning, including personal characteristics and experiences, impacts access to respondents, the nature of the researcher‐respondent relationship, and the way data are interpreted and translated into a study's findings and conclusions.^40^ Recognising and addressing this positioning as an inherent part of the study increases rigour,^41^ especially in practitioner‐based research.^42^

The multidisciplinary research team was diverse. WK, AP and RB are involved in drowning prevention and coastal safety, including research. WK and RB have professional and volunteer ocean lifeguard/lifesaving experience and have designed and delivered beach safety education programs in various settings. AP is a drowning prevention and public health researcher. DA has expertise in applied education research including curriculum design and evaluation.

### 2.6 Ethics and consent

This project was approved by the UNSW Sydney Human Research Ethics Committee under protocol HC210153 and the New South Wales Department of Education via the State Education Research Application Process (SERAP) under reference number 2021098. Lifeguards and beach safety experts provided informed consent for the expert feedback survey and LMCC lifeguards provided their consent to participate in the facilitated lifeguard workshop. For student focus groups, the high school principal and head physical education teacher provided administrative approval, and both parents/guardians and students provided written consent for student participation.

## 3 RESULTS

### 3.1 Expert survey and workshop

The expert survey collected 11 responses (three researchers, seven LMCC lifeguards, and one program administrator) with a median response time of 35.5 minutes. Considering the first draft of the program as a whole, experts felt the amount of content was sufficient (mean score 4.7 [SD: 0.9]; 1—*Major gaps*, 7—*Too much information*) and that the design was good (mean score 5 [SD: 0.9]; 1—*Design needs more work*, 7—*Excellent design*), but did not assess the program as being ready to deliver in schools (mean score 4.1 [SD: 1.0]; 1—*Needs significantly more work*, 7—*Ready as is*). The Likert content scores for individual slides ranged from 3.5 to 5.9 with a mean of 4.9 (SD: 1.4), and Likert design scores for each slide ranged from 3.7 to 5.7 with a mean of 4.9 (SD: 1.4).

The expert survey results served as the framework for discussion in the Workshop, which included 11 participants: eight lifeguards, one lifeguard supervisor, one program administrator and one manager, all employed by LMCC. In the workshop, group scores for each component of the program were shown on a screen along with anonymous suggestions and comments, priming and initiating discussion. Lifeguard educators remained engaged with the material throughout the workshop, expressing interest in the opportunity to have a role in building the school‐program they would be responsible for delivering.

Participants were comfortable sharing their thoughts and providing constructive criticism of the first draft in the workshop environment. Using survey results as a guide helped participants overcome factors that might have prevented them from sharing their thoughts and opinions. Survey responses from managers, supervisors and researchers were included, but not identifiable in the mean scores and suggestions, which allowed for open dialogue and productive disagreement from lifeguards who ultimately had the responsibility of delivering the program in schools.

A major suggested revision was to teach through videos over static slides whenever possible. For example, the first program draft included a 2‐minute video with an explanation of rip currents followed by several static slides explaining what to do in emergency situations. These were replaced by 10 different short videos, and workshop participants collaboratively drafted talking points for each video that collectively covered content that was previously included in static slides. Other significant changes inspired by the workshop included an additional explanation on what lifeguards do and how they can help people; revised content on what to do in an emergency replacing a section on bystander surfer rescue with information on cardiopulmonary resuscitation (CPR), which is more applicable to a broader group of people; and modified content on “inappropriate beach behaviour” re‐organised to a section on “respecting the beach, yourself and others.”

### 3.2 Student focus groups

A total of 26 Year 7 students (12 female, 14 male) participated in focus groups. Results from the focus groups are reported below: first with a description of the student's beach visitation practices, followed by a presentation of four themes relevant for the design and delivery of beach safety education programs for this age group, and finally a short summary of other recommendations made directly by the students.

#### 3.2.1 Beach visitation

Student focus group participants from this coastal area in New South Wales reported frequent beach visitation with most commenting that they go to the beach every day in the summer holiday period, some “*two or three times a day if possible*” (Female, Participant 19). Respondents reported varying levels of permission from parents regarding going to the beach. Some had been going without their parents for several years, mostly with older siblings or other family, but most reported only recently being allowed to visit the beach without adult supervision. Some said they were not yet allowed to go by themselves. There was student recognition that as they get older, they would increasingly be visiting the beach with their friends and not their parents, one student commenting: “*Yeah, next summer we will be more independent*” (Male, Participant 8).

For those students who reported going to the beach frequently with parents, decisions about when and where to go were made largely by the adults. However, when students visit the beach without adults, the decision about which beach to visit was driven by activity and proximity to where they lived. Several students cited choosing one beach over another based on what they wanted to do: one beach was better for surfing, another for swimming and hanging out with friends, and another allowed dogs. As nondrivers, the participants agreed that beach choice was heavily influenced by where they could get to on bikes, skateboarding or public transit. Of note, no participant cited choosing a beach based on safety factors, for example if lifeguards or lifesavers patrolled the beach.

#### 3.2.2 Theme 1: Friends are a driving force for behaviour

Students conveyed concern for wellbeing of their friends, indicating they would act and make decisions accordingly. Several expressed feeling responsible to watch out for their peers at the beach:

“One of my closest friends is not a strong swimmer so like on the big waves I've got to like make sure she's alright when we come up because she gets a little bit nervous.” (Female, Participant #23)

On making safe decisions: “It's out of pure care for your friends because, like, I care a lot about my friends … and I just want them to all stay safe.”

(Female, Participant #26)

When something is not right, or potentially dangerous, some participants were adamant that they would say something. Others admitted peer pressure makes it difficult to do the right thing, and that sometimes they hesitated to speak up for themselves in a situation that might be unsafe. This is evident in the response of one of the students:

“Well, I feel like if you're just with your friends and all your friends are doing it, it is so much pressure.” (Female, Participant #26)

Notably, a nondominant narrative of individual responsibility emerged in one of the male focus groups. In a discussion on what happens in unsafe situations at the beach, in this case large surf and strong rip currents, one student commented:

“I reckon people should know what they can do and their skills so they make their own judgement if they can go out there and catch that wave and stuff.” (Male, Participant #11)

#### 3.2.3 Theme 2: Knowledge and awareness do not always translate to safe behaviour

Focus group participants were knowledgeable about risks present at the beach. They demonstrated an understanding of dynamic and changing conditions, but expressed mixed attitudes on safety and reported limited safety behaviours. Students clearly understood that things go wrong at the beach. They were aware of hazards, and all knew or had heard of someone who had been seriously injured at the beach; many had personal experiences of getting hurt themselves or being present when someone else had. For example,

“Once time around Newcastle someone broke his neck and now he's a paraplegic, and I just know that like anything can happen … you can get hurt.” (Male, Participant #5)

“Yeah, I broke my arm when I was younger at the beach from a wave.” (Male, Participant #8)

They also displayed a clear understanding that safety at the beach is nuanced, agreeing that it is not always safe and not always dangerous. Students provided context and shared their thoughts on changing risk levels, generally concluding that one's safety at the beach depends most often on conditions and activity. Students explained that:

“It all depends on what the surf is. If it's a flat day …. I would consider it safe.” (Male, Participant #3)

“I think you just gotta be smart about it. If it's a big [wave] day you don't go in.” (Female, Participant #22)

“It depends on what you're doing. If you're doing something stupid, then yeah it's definitely dangerous.” (Male, Participant #6)

Despite evident knowledge and awareness of the risks, students admitted that they do not really think about safety when they go to the beach,

“Well, I don't, yeah I'm not really thinking about safety—I just mess around with my friends.” (Male, Participant #4)

However, they agreed that people in their age group probably should be thinking about safety more at the beach:

“Teenagers are probably the age where you're the most careless and reckless you know? So, it is more important that we are thinking about safety.” (Male, Participant #1)

Moreover, this knowledge and risk awareness does not always translate to safer decisions or behaviour. For example, the knowledge that swimming between the flags in areas supervised by lifeguards or lifesavers was the safest option was common:

“We don't need to hear again that we need to swim between the flags.” (Male, Participant #4)

However, implementation of that strategy was mixed: several students did report always looking for the flags and swimming between them with their family and friends, but many said they “*sometimes*” or “*never*” swam between the flags because it was “*too crowded*” (Female, Participant #16) or “*boring*” (Male, Participant #4). The influence of parents and peers on this age group was also apparent with one participant reporting that she usually went to the beach with her father, who “*knows a lot about the ocean and never swims between the flags*” (Female, Participant #19), and another commenting: “*me and my friends will go [to the beach], like well they get to choose which beach, and they say unpatrolled so that's where we go*” (Male, Participant #2).

One discussion point in the focus groups sessions related to a key message in the beach safety education presentation: stopping to assess conditions and look for rip currents before getting in the water. For several participants, getting in the water quickly after arrival at the beach was a priority. Some said they would consider stopping to look for rip currents, but clarified they “*wouldn't spend too much time*” (Male, Participant #7) and would “*just want to jump in*” (Male, Participant #14).

#### 3.2.4 Theme 3: “Give it to me straight”—levelling up from “what” to “how and why”

A reoccurring conversation in the student focus groups related to the self‐perception of being “*older now*,” especially as the conversation moved towards gathering input on beach safety education programs in their school. Students saw themselves as more mature and independent, emphasising they were ready for more advanced content. They thought that beach safety education programs targeted at their age group needed to go beyond telling students what to do, that more sophisticated explanations and reasoning behind safety advice would be more effective. One student explained that:

“You have to not only tell them what they should do but also why. The why is very important, like for swimming between the flags. An 8‐year‐old you can say ‘just swim between the flags’, but now for people in our year you need to tell them why they should be swimming in between the flags.” (Male, Participant #3)

Students also expressed a desire for information on what they could do in an emergency at the beach, either to help themselves or others. This was summarised by a student as follows:

“They should tell us how to do something if something does happen. All through primary school when we had lifeguard talks, all they would talk about was rips and stuff. And I'm like ‘I already know that’ like I've heard that before, why do I need to learn this stuff over and over and over again? But they still tell us. I reckon they need to tell us the big stuff like what's going to happen and how you can assess it and how you can help yourself to get out of a situation.” (Male, Participant #11)

#### 3.2.5 Theme 4: Conflicting thoughts on stories and statistics

Focus Group participants mostly agreed that stories were a powerful tool to convey messages and connect with students, but disagreed on the impact and effectiveness of using true stories of injury or death as a motivator. While several students recommended lifeguards tell “*scary stories*” about people getting hurt to get the attention of their peers, many thought that approach would not work for people their age. Simply put,

“If you show them too much it turns them off. They just don't want to go there anymore, so you have to be careful [with] what type of things you show or share.” (Female, Participant #20)

There was a similar divergence of opinions on the usefulness of facts and statistics: some students thought their friends needed to hear numbers about drowning or beach injuries, and others warned that it could be boring and might cause some people to tune out. Similar to Theme 2, students were largely aware of things that go wrong at the beach: they see it on the news, hear it from their parents and friends, and it has not altered the way they think or act. These focus group participants questioned if spending limited time in a school beach safety presentation on a story about serious injury or death was worthwhile.

#### 3.2.6 Student recommendations for high school beach safety education

Students provided several recommendations for lifeguards who deliver beach safety education programs in schools. In relation to program style and feel, students recommended that lifeguards present with a relaxed and casual approach (“*don't be too serious*”; Male, Participant #1), use humour (“*funny is memorable and will stick with [students]*”; Male, Participant #12), engage students in activities where possible (“*If you don't get people involved, they won't remember a lot of stuff*”; Female, Participant #17), bring in lifeguard equipment to show and/or do some sort of demonstration, and make sure the presentation included videos (“*[videos] are more fun, good to mix in with slides*”; Female, Participant #25). In terms of program content, students advised that lifeguards should frame their messages in the context of students going to the beach without adults, including tips and guidance on handling peer pressure situations:

On peer pressure situations: “*Some tips on how to approach it, like how to talk to friends who are doing something wrong*.” (Female, Participant #18)

Additional content recommendations related to sun safety, cliff and jetty jumping, stingers, CPR/First Aid, how to help if something goes wrong, and taking care of the environment. The shift on focus of the program is explained by a student below:

“I reckon in primary school they've done enough about what to do to help ourselves, but I reckon in high school we should learn what to do if you see someone else in [a dangerous] situation.” (Male, Participant #9)

## 4 DISCUSSION

The lessons learned from this mixed‐methods study of an expert and student co‐designed school‐based beach safety education program should inform future efforts. The co‐design process itself proved to be an efficient and inclusive model for program development, and insights on the beach experiences and safety attitudes of pre‐teens and younger teenagers, our priority population, were of immense value for refining and tailoring the program's messages. Below, we discuss how results from the different components of this study informed changes and updates to our specific program, within the general context of promoting safe beach‐related decision making and behaviour. While the lessons learned from this project are particularly important for beach safety practitioners designing school beach safety education programs for Australian coastal communities, they also have implications for anyone working in safety promotion and injury prevention education.

The co‐design process was a valuable methodological approach on multiple levels. First there was direct benefit from including expert voices from different fields (researchers and ocean lifeguards) in the survey development phase and the expert workshop. Previous researcher‐teacher co‐design work has identified the value of teachers' practical experience in the classroom for curriculum development^43^; similarly, the perspective of lifeguard educators challenged some of the approaches conceived by researchers or their supervisors. Moreover, while the actual suggestions and feedback from lifeguards were important, the fact that their voices and perspectives were included and taken seriously alongside researchers, supervisors, and managers served to build trust and buy‐in for the program, which is critical as they are the individuals responsible for delivering it.

Second, student input provided insights that made researchers and lifeguards consider the program in ways that would not have been possible by engaging with experts alone. While specific student recommendations on program format and content were helpful, understanding more about their experiences at the beach and with in‐school beach safety education highlighted the need for a pedagogic approach designed specifically for them. The previous beach experience of this age group was foundational to their understanding of, and attitudes towards risk. The student focus group portion of the co‐design process allowed us to unlock some of those experience‐informed beliefs and attitudes and caused us to re‐think how a beach safety education program should be structured for this age group.

Pre‐teens and teenagers in this Australian coastal community were knowledgeable about beach hazards and aware of the inherent risks, but displayed mixed safety attitudes and self‐reported safety practices. This is not necessarily surprising, similar findings were observed in Australian university students^44^ and older teenagers (ages 15‐18) in New Zealand,^21^ and the student focus group participants came from a surf‐culture oriented coastal community where youth beach programs such as “Nippers” and activities including surfing, paddling and ocean swimming are popular. Still, some emerging themes from the focus groups made us pause to reconsider our program's messages, for example, the fact that students (i) knew swimming between the flags was the safest option, (ii) did *not* want it to be included in their education program, and (iii) still reported swimming in unpatrolled areas.

Behavioural change approaches and health theory may help explain the disconnect between knowledge and behaviour, and have previously been employed by beach safety researchers to improve understanding of motivational factors and barriers.^21^, ^44^, ^45^ We found the Theory of Planned Behaviour^37^ a useful aid for operationalising findings from the student focus groups into actionable improvements to the beach safety program. Relying on this framework helped us understand students' experiences in terms of behavioural beliefs related to their perceptions of risk and attitudes towards safety practices; normative beliefs related to *whose* opinion mattered, which for this age group was largely in flux; and control beliefs of their perceived ability to help in an emergency or make a safe decision while facing peer pressure.

Descriptive and injunctive norms, the perceptions of what others do and approve of, are powerful drivers of behaviour,^46^ and offer opportunities to craft meaningful safety messaging for a targeted audience. While parents were still an important authority and source of information for many students, the influence of friends was growing stronger and the collective responsibility to watch out for one another was apparent. We therefore re‐framed several key messages in the education program from being focused on the student themselves (eg, swim between the flags to keep yourself safe) to positioning the impact of one's decisions on their friends (eg, swim in between the flags to keep all your friends safe). Other water safety messaging campaigns have also employed this approach^47^; evaluating if this strategy is more effective across different age groups and demographics is an important next step.

Similarly, we revised some elements of the educational program to address specific control beliefs, namely the student's perceived ability to make a safe decision at the beach. In line with previous literature,^48^ we found that peer influence to engage in unsafe activities, such as cliff jumping or swimming at unpatrolled beaches, presented as an important factor that impedes safe decision‐making. Adolescents engage in riskier behaviour when in groups^49^ and resistance to peer influence is limited for those aged between 10 and 14 years of age.^50^ However, the presence of peers is multidimensional; friends can encourage or discourage certain behaviours,^51^ which, linking back to social norms, provides an opportunity to craft safety messages. With this insight, the lifeguards now discuss risky beach behaviour in parallel with individual autonomy, reminding students that they have the power to make their own decisions, they should “take five seconds to think for themselves” when they are feeling pressure from others.

This study had some limitations. Student participants were from a coastal high school in a socio‐economically advantaged suburb where 85% of the population was born in Australia and 90% speak English at home.^52^ The goal of qualitative research is not to generalise,^31^ however, learning about the experiences and perceptions of adolescents who live in inland or disadvantaged areas, speak a language other than English at home, or identify as members of nondominant cultural or ethnic communities may lead beach safety educators to design programs differently. This study provided valuable insight for our specific purpose and suggests that similar co‐design processes should occur regionally or with an identified specific priority population, including those for adults. To this point, we did not gather socioeconomic, cultural, language or additional demographic information from focus group participants, which would have aided analysis and our understanding of this population. Future research and co‐design efforts in this space should prioritise collecting this information and considering these factors in analysis and development of programs.

The group effect is an advantage of focus groups where interaction between participants can produce rich data and insights^31^; but it could have prevented some participants from speaking up, creating the potential for us to miss some information. In future work, triangulation of these data with multiple methods, such as anonymous surveys or in‐depth interviews, would be an important consideration. Future qualitative work that engages in purposeful sampling^30^ of young people who have engaged in dangerous activities or those in early adolescence who have experienced adverse beach events would serve to further develop a theory explaining motivators of behaviour for these groups, which may provide a more robust foundation from which to design education efforts.

## 5 CONCLUSION

The co‐design model that sought systematic feedback from a group of experts, including researchers and ocean lifeguards, and end user students was helpful for refining the school‐based beach safety program. The student focus groups identified several facets of this age group's beach experience that prompted revision of the education program, primarily the framing of program content and safety messages. The students were regular beach users who were knowledgeable and mostly aware of beach related hazards and risks, but did not always make the safest decisions. Future education efforts for this population require focus on promoting safe behaviour vs informing students about hazards and risk. This study illustrates the value of adopting co‐design processes for all beach safety programs, school or otherwise. Structured consultation with the priority population should become standard practice for development and application of future beach safety programs across all ages, both in Australia and globally.

## FUNDING INFORMATION

This study was funded by the Science Industry Network Seed Fund (#RG210654, #RG210655) which included financial support from UNSW and the Lake Macquarie City Council.

## CONFLICT OF INTEREST

The authors declare no conflict of interest.

## ETHICS STATEMENT

UNSW Sydney Human Research Ethics Committee: HC210153. New South Wales Department of Education (SERAP): 2021098.

## Supporting information


**Appendix S1** Supporting Information

## Data Availability

The data that support the findings of this study are available on request from the corresponding author. The data are not publicly available due to privacy or ethical restrictions.
