# Supplementary material for: Lessons learned from co‐designing a high school beach safety education program with lifeguards and students
Source: Health Promot J Austr. 2022 Sep 25;34(1):222–31. doi: 10.1002/hpja.664 (PMC10087910; doi:10.1002/hpja.664)
Supplement: Supplementary file 1 — Appendix S1 Supporting Information [file HPJA-34-222-s001.docx]

**Australian Beach Safety Education Evaluation Project**

**Student Focus Group Guide**

Welcome

- Introduce moderator(s)
  - Name
- Introduce topic
  - Beach safety, more specifically, beach safety education for high schoolers
  - Why is this important?
- This discussion is for a research project
  - This discussion is part of a larger research project that is trying to improve beach safety education in high schools, your input is very valuable

Guidelines

- No right or wrong answers, only different points of views
  - Please feel free to share your perspective, even if it is different than someone else's
  - You don't have to agree with other people, but please be respectful as other people share
- We are audio recording this session
  - We are recording because people often say helpful things and it is difficult to write everything down quickly
  - We will write everything from the recordings down later, and we will not use your name in any part
- My role as the moderator
  - Help guide the discussion
  - Will call on people as they raise their hand
- We are on a first name basis
  - We will use first names during our discussion
  - Please use your name tag we have provided

Opening questions:

- - Have students share:
    - Who goes to the beach in the summer?
    - What do like about the beach? Why do you do at the beach? WHY?
    - What is your favourite beach, WHY?
      - What goes into where you decide to go?
      - How do you get to the beach?
    - Last summer, who did you go to the beach with?
      - Parents?
      - Family?
      - Just friends?
        - Do your parents talk to you about safety if you go alone?
    - Your day at the beach (What are the beach priorities?)
      - When you get to the beach, toes in the sand, what is the first thing you think about or do?
        - Friends? Where to set up? What about risk/safety?

Key questions:

**Is the beach a safe or a dangerous place? (raise your hands)**

- Why is it safe/ dangerous?
- Confounding / opposing views and facts.
- What are the things you are most concerned about a the beach? Why?
- How concerned are you? If not, why?
- IF low concern -> Do you think people in your age group are thinking about these things?
- If High concern -> what do you do to keep yourself safe?
- Should safety be a higher priority in your age group? Why?

**How would your peers be motivated to prioritise safety at the beach?**

- What would make you or your friends think about safety when at the beach?
  - Stories?
  - Statistics?
  - Science about hazards?
- Where would you get this info?
  - Internet?
  - Is the school the right place?
- Who do you trust?
  - Lifeguards?
- When should we talk to you about this? Winter? Right before summer?

**Now we are going to talk about lifeguards coming into your school to give beach safety talks.**

- Have you had lifeguards give beach safety talks in school before?
  - What did you like or not like? What worked? What didn't?
  - What kind of topics should they talk about?
  - What would be engaging and interesting?
- Are there different beach safety topics for primary school and high school students?

Back up questions:

**What do you think lifeguards should talk about in beach safety presentations for high school students?**

Probing Questions:

- What topics would you like to hear about from the lifeguards?
- What is the best way to get high schoolers to follow through with acting safely on the beach?
- What is the best way to make the presentation engaging?
- Are there different beach safety topics for primary school and high school students?

**How should the lifeguards make their presentation memorable and engaging?**

Probing Question

- What would make their presentation interesting?
- Have you had good or bad guest speakers come in before? What made them good/bad?

Ending question

**Out of everything we discussed today, what advice would you give to lifeguards who are going to give beach safety presentations in high schools?**

Closing question

**Have we missed anything? Is there anything else anyone would like to add?**
